# A Case of Aneurism of the Abdominal Aorta, with Notes of Post Mortem Examination

**Published:** 1884-07

**Authors:** Mark H. Sears

**Affiliations:** Leadville, Col.


### ARTICLE IV.

### A CASE OF ANEURISM OF THE ABDOMINAL AORTA, WITH NOTES OF POST MORTEM EXAMINATION.

*By* MARK H. SEARS, M. D., *Leadville, Col.*

I was called some time since, to see, A. H., a native of Switzerland, thirty-seven years of age, and a resident of the United States for about ten years; occupation, a miner and its kindred pursuits; of phlegmatic temperament. He stated that about two years ago he first began to complain, but that his symptoms were obscure and uncertain. About eighteen months ago he began to have some pain in the umbilical region. At the same time, or soon after, he noticed some induration and a sensation of fullness, which had since gradually increased.

I found his pulse small and thready, about 100 per minute; temp., 100 F.; respiration about 25 per minute; skin dry and harsh; capillary circulation sluggish; bowels fairly active, and alvine discharges apparently normal; appetite capricious, evincing some desire for the animal broths; assimilation poor; mentality good, answering without hesitation questions addressed to him. Complained of some pain in the left lumbar region of a dull and heavy character. A few days before I saw him, he had complained of severe neuralgic pains in the course of the anterior crural nerve and its branches. These symptons had been relieved by morphiæ sulphas in suitable doses, and had ceased to recur when I visited him. These were the only symptoms of spinal origin which occurred in the history of the case.

The patient had also been "leaded" several times—this is the common expression for lead poisoning among the miners of this region. Upon examining the abdomen I found presenting in the lower portion of the left inguinal region, an indistinctly fluctuating tumor of considerable firmness. It extended from midway of Poupart's ligament, to a point upon the linea alba two inches above the crest of the pubes.

There existed in it a distinct thrill with each ventricular contraction, that expanded the walls of the tumor equably with each pulsation. Pursuing the examination upward, I found in the upper portion of the left inguinal and lower portion of the left lumbar regions a hard, resisting, non-fluctuating body, situated subcutaneously. Subsequent information obtained post mortem, proved this to be the left kidney. Continuing the course of the tumor, which had again resumed its firm though fluctuating character, I traced it upward through the region last mentioned into the left hypochondriac region, where its outlines were partially lost beneath the floating ribs.

Throwing the patient upon his right side, there appeared upon the back of the left side a circumscribed enlargement of the size of a large orange. It commenced at a point about one and a half inches to the left of the tenth and eleventh dorsal vertebra, extending around the side anteriorly.

The integument covering it was injected, cyanosed, nearly devoid of sensation, and dotted at intervals with minute points of ulceration. The physical characteristics were the same as those presented by the tumor in the abdomen. They were better marked, however, as the tissues covering the enlargement were being absorbed rapidly and consequently its covering thin. Percussing it, the resulting note was heavy and dull, as though the tumor were solid. As the post mortem revealed one or two inches of semi-organized fibrin lining the cavity of the growth, it explains the non-appearance of a percussion note more tympanitic.

Auscultation gave greater satisfaction, and revealed the aneurismal "bruit," although somewhat indistinctly. The second sound of the heart was well defined in the tumor. The cavity of the chest presented the physical symptoms of hydrothorax of left pleura; while in the right lung the normal vesicular murmur and resonance existed only in the lower portions. Heart's action was weak and uncertain, apex pushed forward and to the left, impulse in fifth intercostal space, area of præcordia diminished in size.

From this association of symptoms I diagnosticated "Aneurism of the Abdominal Aorta."

The patient died May 12th, 1884, probably from exhaustion, there being no rupture of the aneurismal cyst.

Post mortem examination three hours after death.

Body greatly emaciated, of about 100 lbs. weight, some icterus.

Same tumefactions as had existed in life present; but now, instead of being firm and unyielding, they are soft and compressible.

First line of incision passed from pubes to top of sternum, intersected by the usual crucial incision in abdomen. Flaps reflected back, Stomach of normal size, shape, etc. Position entirely changed. The greater curvature lay immediately in contact with the abdominal parietes, the lesser curvature toward the vertebral column, and embracing the renal vessels of the left kidney, whose position will be described presently. The cardiac and pyloric orifices, which were respectively superior and inferior, were on nearly a direct plane.

At the left of the stomach, covered by the abdominal muscles, great omentum, and partially in the inguinal and lumbar regions, as previously mentioned, lay the left kidney. It was enclosed in a capsule of adventitious tissue—possibly the elements of the normal fibrous capsule pathologically enlarged and added to by the results of a very low chronic inflammation, whose results were everywhere apparent in the great number of adhesions.

After carefully removing this capsule, the kidney and renal vessels were exposed. The latter were elongated and passed beneath the lesser curvature of the stomach to their respective origins. Removing the kidney and cutting into it, both the cortical substance and medullary portion were found infiltrated with fat—fatty degeneration.

Liver, healthy, but rather small for a person the size of the subject.

Spleen, normal, but somewhat flattened between the walls of the abdomen and the walls of a large tumor which now presented. The small intestines had been forced into the cavity of the pelvis and right side of the abdomen.

The tumor, which was ten inches long, lay with its base pressing upon the external iliac artery, while its large bulbous apex pressed into the posterior hypochondriac region on the left side and formed the large protuberance externally on the back.

It embraced both sides of the bodies of the four upper lumbar vertebræ with their transverse processes, and the bodies of the eleventh and twelfth dorsal vertebræ. So firm were the adhesions that it was not practicable to remove it in its entirety. In this event I deemed it best to evacuate the contents if possible. I made an incision into it, and after passing through one and one-half inches of organized fibrinous material lining the sac, I reached a cavity, and verified the diagnosis of aneurism which had been made

The contents of the cavity were clotted blood, which with the fibrinous material was removed. The opening into the aorta was one and one-half inches below the cœliac axis, was three-quarters of an inch wide, and one and one-quarter inches long. The wall which had existed between the aneurismal cavity and spine had been absorbed, so that the bodies of the vertebra were exposed to the direct action of the blood and pressure of the aneurism. Never were the effects of continued pressure better exemplified. The transverse processes of the first, second, and third lumbar vertebræ were completely destroyed on both sides; those of the fourth lumbar, partially; also the bodies of the several vertebræ mentioned, together with those of the eleventh and twelfth dorsal, to nearly their center, and in one instance an opening into the spinal canal. It is worthy of remark that there was no evidence of functional derangement of the spinal cord in life other than that of the crural neuralgia mentioned in the first part of this article. The lesion would seem severe enough to have interfered with the integrity of the spinal canal, and produced pressure upon the cord with its obvious results.

The sac with its contents having been carefully removed, was found to weigh 6½ lbs. av.

There was no rupture in its walls, which were one-quarter of an inch thick.

The examination of the heart revealed fatty degeneration. The left lung was collapsed and bound down by old pleuritic adhesions; while the pleural cavity contained about three pints of serum—much of it post mortem, I presume. The upper lobe of the right lung was consolidated and contained a small abscess.

In conclusion. There was neither history nor evidence of syphilitic taint, none of violence. The disease occurred in the person of a strong middle-aged man, whose only ailment previous to this fatal disease, had been several attacks of acute lead poisoning, which probably had saturated his system. In the absence, then, of evidence to the contrary, I shall ascribe his aneurism to chronic lead poisoning.

